# Using debriefing and feedback in simulation to improve participant performance: an educator’s perspective

**DOI:** 10.5116/ijme.55fb.3d3a

**Published:** 2015-09-25

**Authors:** Claire L. Burns

**Affiliations:** 1 Department of Medical Education, Royal Bolton NHS FT, Bolton, UK

**Keywords:** simulation based medical education, feedback, debrief, simulation debrief

## Background

As a clinical skills educator I am involved in the delivery of simulation based education for UK trained doctors in their first year post qualifying (Foundation Year 1 [FY1]). I have an interest in the use of feedback and debrief as a tool to improve performance through reflection.

When relating simulation to Kolb’s^1^ learning cycle it is evident that taking part in the simulated scenario only accounts for the concrete experience component. Debrief and feedback accounts for the reflective observation, abstract conceptualization and active experimentation components of the learning cycle by helping participants make sense of the simulation scenario and reflect on their practice to improve future performance.

### Preparation for simulation

A simulation event took place in a hospital skills laboratory set up to resemble a patient bed space.  All simulation scenarios involved the management of an acutely unwell patient. Each participant was an FY1 doctor and all participated in a simulation scenario followed by feedback from both faculty and the other participants attending the event.

Four participants attended the event in total. This small group size allowed for debate and discussion as well as helping students to feel relaxed and promoting interaction between the participants. All participants had completed their training in the UK so it was assumed they already had a threshold of knowledge and skills as documented by the General Medical Council (GMC).^2^

Prior to the simulation event individualized scenarios were developed relevant to each participant’s current placement as it is important for the simulation to be appropriate for students’ needs.^3^^, ^^4^ It could be argued that by only focusing on what it is felt students “need to know” they are not experiencing a full breadth of learning. However, I consider that the scenarios delivered to the students are common emergency scenarios that could potentially be experienced across all disciplines of medicine.

Learning outcomes that fulfilled the requirements of the GMC^5^^, ^^6^ were usedas a basis for developing the simulations. Clear outcomes were set for the event so participants knew what knowledge; skills, attitudes and behaviours needed to be demonstrated.^3^^,^^8^ The outcomes included both the technical and non-technical aspects of care delivery. Both of these aspects are imperative in delivering safe patient care ^5^^-^^9^ and are focused on critical thinking and problem solving.  Discussing the learning outcomes at the beginning of the event enabled exploration of the importance of non-technical skills. In the future, I plan to ask the students what their objectives for the session are to increase participation, motivation and performance.^10^ The HEA^11^ recognizes that student centred learning increases student confidence and excitement about the subject.

### Delivering the simulation

On the day of the simulation, students were given an overview of each of the faculty members’ roles and information regarding the manikin’s limitations and equipment being used. Identifying limitations before starting the scenarios improved fidelity^3^ as the faculty didn’t have to interrupt the scenario to acknowledge constraints.

Providing a pre-brief at the beginning of the session was useful and helped to facilitate reflective practice by preparing students for the discussion at the end of their scenario and making them aware of how they would receive their feedback.^12^ It also alerted the students that they were equal partners in the feedback process and triggered internal feedback.^12^^, ^^13^

Each student was allocated a scenario and acted as the team leader whilst faculty acted as other members of the healthcare team. During the scenario the faculty observed the students’ so that feedback could be given. However,direct observation does not facilitate in-depth exploration of clinical reasoning or problem-solving abilities.^14^ In future sessions a member of the faculty will act as a medical student to question the participant and determine their underpinning knowledge.

### Debrief and feedback

After completing the individual scenarios, oral feedback was given to students by the faculty and their peers. Group feedback and peer learning are all effective assessment for learning tools.^11^ Individuals can learn a lot through the experience alone but specific feedback will maximise learning.^15^^, ^^16^

Waiting until completion of the scenario before giving feedback allowed the participants to self-reflect and make sense of what had just happened. Facilitators and peers were then able to discuss the strengths and weaknesses of the student’s performance without interrupting the scenario and decreasing scenario fidelity and allows participants to discuss the consequences of their actions.^15^

Feedback on the participant’s performance is the most important feature of simulation education as it produces long lasting learning and allows the student to develop a deep insight and reflection about their performance as well as slowing the decay in knowledge.^3^

During debrief students discussed any emotions that they had about the simulation scenario as well as reflecting on and exploring their decision making processes. Giving oral feedback to students enabled the faculty to be flexible with their questioning, allowed an immediate response from the student and permitted clarification of any misunderstandings.^17^ However, oral feedback does not allow the person giving feedback time to reflect on the student’s performance.^18^

A criticism from one of the participants was that they would have liked written feedback for portfolio evidence. One option would be for the faculty to meet after the event, discuss each student’s performance and then email individual feedback. However, this option would be time consuming and may not be feasible.

When giving feedback, the first question the student was asked was: “How do you feel that went?” This facilitated self-evaluation which is essential to reducing the emotive impact of feedback.^13^ Facilitating self-evaluation will also promote the student to function in a reflective mode in their daily practice. However, we all hold biases in the way we judge our own performance.^13^ Self-evaluation relies on the student to be self-aware and effective at critiquing their own performance;^13^ a skill not always present. Self-evaluation alone is inadequate for performance improvement,^19^ it needs to be facilitated by skilled evaluators who can change their questioning strategy appropriately to ensure student understanding.^17^

Overall participants evaluated the simulation event as a valuable learning experience that gave them a chance to apply their theoretical knowledge to simulated reality and made them aware of the national and local guidance available to them. However, feedback was identified as an area for faculty development. One student asked for “more strict feedback” This feedback itself is somewhat unhelpful due to its vagueness. Another student asked that the “feedback sandwich” be “more strictly enforced”. I have never been a devotee to the feedback sandwich as I find it predictable, patronising and a wasted opportunity to discuss the meat of the issue and improve student performance. For feedback to be useful it needs to lead to action which will improve the student’s performance.^1^^,^^13^^,^^16^^,^^19^ Feedback can only do this if it identifies specific areas for development and supports the learner in identifying strategies to bridge the gap between current and desired performance.^5^^, ^^16^

The simulation event presented valuable learning for the faculty. Most student errors were human factor errors. For example, guidelines were either not used or not followed correctly and communication was often poor leading to delays in patient treatment. In future sessions more emphasis will be put on human factor training and a structured model of debrief will be used.

### Conflict of Interest

The author declares that she has no conflict of interest.
